# Relationship satisfaction in lesbian and heterosexual couples before and after assisted reproduction: a longitudinal follow-up study

**DOI:** 10.1186/s12905-014-0154-1

**Published:** 2014-12-12

**Authors:** Catrin Borneskog, Claudia Lampic, Gunilla Sydsjö, Marie Bladh, Agneta Skoog Svanberg

**Affiliations:** Department of Women’s and Children’s Health, Uppsala University, S-751 85 Uppsala, Sweden; Neurobiology, Care Sciences and Society, Karolinska Institutet, S-141 83 Huddinge, Sweden; Division of Obstetrics and Gynecology, Department of Clinical and experimental Medicine, Faculty of Health and Science, Linköping University, Linköping, Sweden; Department of Gynecology and Obstetrics in Linköping, County Council of Östergötland, Linköping, Sweden

**Keywords:** Relationship satisfaction, Lesbian couples, Sperm donation

## Abstract

**Background:**

More and more lesbian couples are planning parenthood through donor insemination and IVF and the number of planned lesbian families is growing in Sweden and other western countries. Research has shown that lesbian couples report as much overall satisfaction in their relationships as do heterosexual couples. However, although parenthood is highly desired, many parents are unaware of the demands of parenthood and the strain on their relationship that the arrival of the baby might bring. The aim of this study was to compare lesbian and heterosexual couples’ perceptions of relationship satisfaction at a three-year follow up after assisted reproduction.

**Methods:**

The present study is a part of the Swedish study on gamete donation, a prospective longitudinal cohort study. The present study constitutes a three-year follow up assessment of lesbian and heterosexual couples after assisted reproduction. Participants requesting assisted reproduction at all fertility clinics performing gamete donation in Sweden, were recruited consecutively during 2005–2008. A total of 114 lesbian women (57 treated women and 57 partners) and 126 heterosexual women and men (63 women and 63 men) participated. Participants responded to the ENRICH inventory at two time points during 2005–2011; at the commencement of treatment (time point 1) and about three years after treatment termination (time point 3). To evaluate the bivariate relationships between the groups (heterosexual and lesbian) and socio-demographic factors Pearson’s Chi- square test was used. Kolmogorov-Smirnov test was used for testing of normality, Mann–Whitney U- test to examine differences in ENRICH between the groups and paired samples t-test to examine scores over time.

**Results:**

Lesbian couples reported higher relationship satisfaction than heterosexual couples, however the heterosexual couples satisfaction with relationship quality was not low. Both lesbian and heterosexual couples would be classified accordingly to ENRICH-typology as vitalized or harmonious couples.

**Conclusions:**

At a follow-up after assisted reproduction with donated sperm, lesbian couples reported stable relationships and a high satisfaction with their relationships, even when treatment was unsuccessful.

## Background

Research into factors that influence intimate romantic relationships has previously been mainly conducted on heterosexual couples. However, the research body on relationships in same-sex couples is increasing. In general it seems that aspects that influence heterosexual relationships also influence same-sex relationships. For example, in a review of empirical studies of same-sex couples in the United States, it was suggested that the similarities between same-sex and heterosexual couples far outweigh the differences, both in relationship quality and the processes that regulate satisfaction and commitment [1].

During the last decades in Sweden, there have been a series of societal changes in order to provide people with the same rights and opportunities regardless of sexual orientation. In 2003, adoption of children was opened up to same-sex couples; in July 2005 assisted reproduction with donated sperm become available to lesbian couples within the Swedish public health care system; and in 2009 the gender-neutral legislation about same-sex marriage was introduced (SFS 2009:260) [2].

The desire to have children is the same to all individuals. To some the desire is very strong, to some it is weaker. Like heterosexual women, many lesbian women have a strong desire for parenthood and want children [3-5]. A planned lesbian family is when two women have opted for motherhood within a lesbian relationship. However, planned lesbian families differ from lesbian families with children originating from heterosexual relationships [6,7]. In the former families the parental composition has changed, and parent and child have experienced divorce and the coming out of the mother [6,7].

The transition to parenthood is the time and psychological process people and couples undergo during pregnancy and the first months after birth; a psychological process changing women and men into parents. According to Lewis [8], who describes the transition to parenthood, it seems that relationships which function well before pregnancy and birth remain good during the baby’s first year. Relationships where the spouses have problems with communication and emotional intimacy seem to be more vulnerable with regard to parenthood [8]. In a study from Sweden of first-time parents experiences of their intimate relationship it was found that, although parenthood is highly desired by the couples, they are unaware of and not prepared for the demands of parenthood and the strain on their relationship that the arrival of the new baby can bring [9].

Research has shown that lesbian couples report as much overall satisfaction in their relationships as do heterosexual couples [10]. Higher educational level of lesbian individuals and the fact that lesbian couples are less likely to have previous children are factors suggested to impact positively on lesbian couples’ relationships [11,12].

Previously we have reported a high relationship satisfaction in lesbian couples [13]. We also found that relationship satisfaction in lesbian women with previous children was lower than it was in lesbian women without previous children.

Donor insemination in lesbian couples [14-16] and fertility treatments [17,18] are known to be stressful and influence on the quality of life of the couples in all kinds of ART. Psychosocial wellbeing, sexual satisfaction and marital relationship are aspects mentioned as being impaired by fertility treatment [17,19-23]. Further, a decrease in relationship satisfaction may affect the transition to parenthood for couples undergoing fertility treatment [24]. The couples relationship during the first year of parenting is particularly vulnerable and many divorces take place during this time [9].

Lesbian couples starting a family through sperm donation treatment are a new and growing [13] group of patients in obstetric and neonatal/paediatric care in Sweden. However, little is known about the intimate romantic relationship of lesbian couples planning a family with children. Unique to lesbian couples is the fact that they are two women planning a family together where one of the parents will not have a biogenetic link to the offspring. Additionally, lesbian couples are a largely stigmatized group and have previously been (unfairly) depicted as having many psychosocial problems [25,26].

The aim of this study was to study lesbian couples satisfaction with their relationship at a three year follow-up after assisted reproduction treatment and to relate these findings to pre-treatment perceptions of the relationship and demographic variables. Another aim was to compare these variables with a group of heterosexual couples undergoing IVF-treatment with their own gametes during the same treatment course.

The following specific research questions were posed:

1. How do the perceptions of relationship satisfaction between lesbian and heterosexual couples at three-year follow-up differ compared to their perceptions of the relationship before treatment?
2. Are the perceptions of lesbian and heterosexual couples’ relationship satisfaction related to demographic variables such as age, level of education and being the treated women or the partner?
3. Are the perceptions of lesbian and heterosexual couples’ relationship satisfaction related to a successful outcome of the treatment?

## Methods

The Swedish study on gamete donation is a prospective longitudinal study aiming to investigate the psychosocial and medical aspects of donor conception. The multi-centre study includes both donors and recipients of donated gametes, as well as a comparison group of heterosexual couples using IVF-treatment with their own gametes. Participants were recruited from all fertility clinics performing gamete donation in Sweden i.e. University hospitals in Stockholm, Göteborg, Uppsala, Umeå, Linköping, Örebro and Malmö.

During 2005–2008, a consecutive cohort of lesbian ART (assisted reproduction treatment) couples at the commencement of sperm donation treatment, were approached for participation. For comparison, heterosexual couples that started standard IVF-treatment with their own gametes at four of the participating fertility clinics were asked to participate in the study. Inclusion criteria were: being able to read and understand Swedish well enough to answer the questionnaire. The longitudinal study consisted of data collection at three time points; at the commencement of treatment (T1), two months after treatment (T2) and about three years after treatment (T3) when a potential child was between 12–36 months old.

The questionnaires at this presentation were distributed by mail together with a prepaid return envelope and a cover letter stating the purpose of the study and guaranteeing confidentiality. The partners in the couples received one questionnaire each. The couples were asked to complete the questionnaire individually. Two reminders to non-responders were sent.

Of a consecutive cohort of 214 lesbian couples (428 individuals) about to receive donor insemination and 212 heterosexual couples (424 individuals) starting regular IVF treatment with their own gametes, 166 lesbian couples (78% response) and 151 heterosexual couples (71% response) accepted participation in the study (T1).

**Figure 1:**
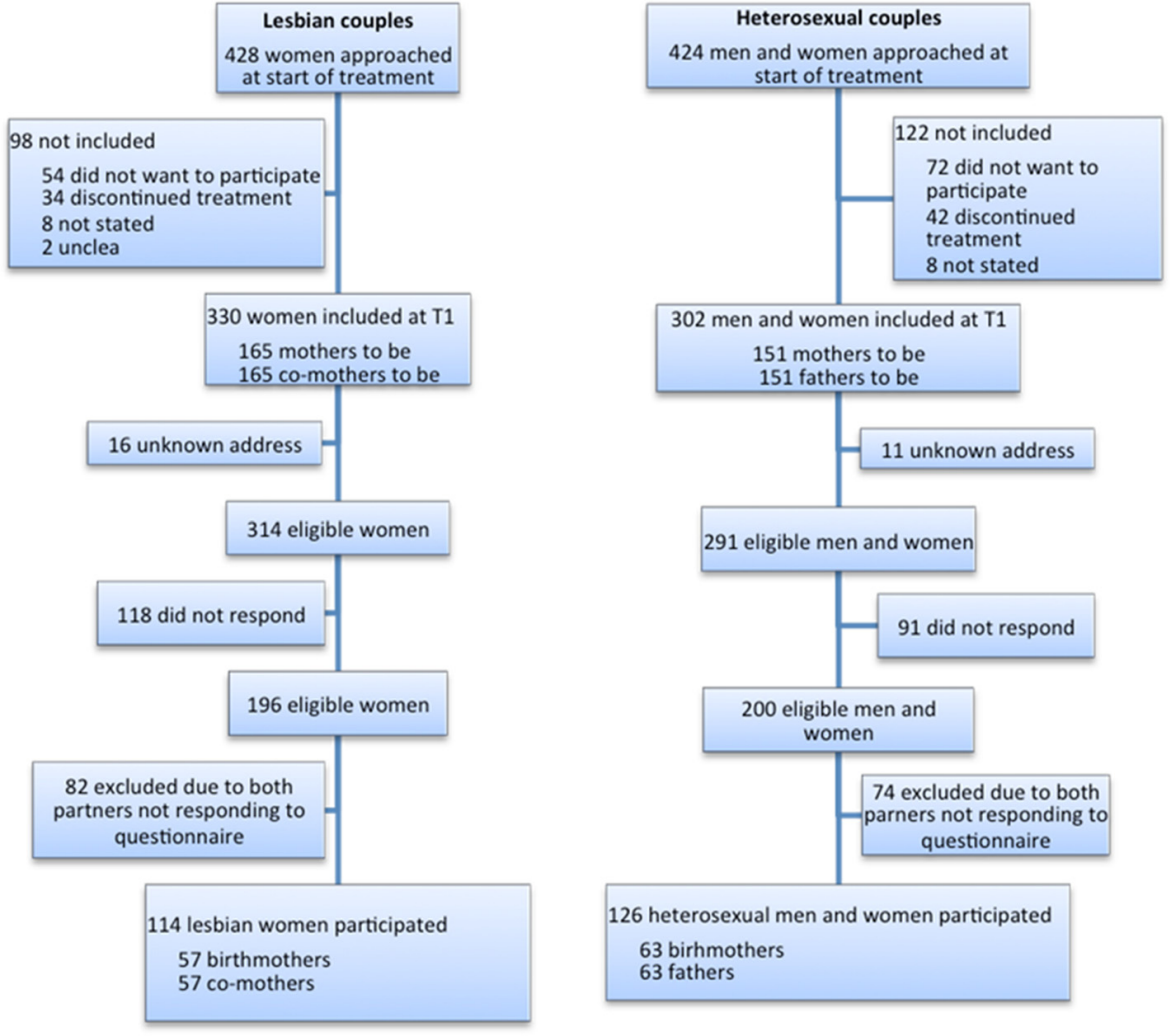
**Flow chart over participants and drop-outs.**

Since one aim of this study was to investigate changes over time in couples’ relationship satisfaction before and after assisted reproduction, only couples that responded to questionnaires at commencement of treatment (T1) and about three years after treatment (T3) were included. Of the eligible couples, 57 lesbian (57 treated women and 57 partners) and 63 heterosexual (63 women and 63 men) couples participated in this study and responded to the questionnaire. Figure 1 displays a detailed description of participants.

Previous studies have shown that the similarities between lesbian and heterosexual couples far outweigh the differences [1,27]. The lesbian and heterosexual couples turn to assisted reproduction due to a strong desire to conceive and to have children. Before being accepted for assisted reproduction the couples undergo a psychosocial investigation and consequently, the couples in this study are psychologically healthy. It is reasonable to believe that, although different in nature, the experiences of distress related to both medical and “social” infertility influence psychological wellbeing. In addition, in this sample, 65% of the lesbian women also underwent IVF-treatment and it is likely that the hardship of IVF-treatment and hormone stimulation are similar to both lesbian and heterosexual women. These aspects of similarities provide a solid foundation for comparisons of changes in relationship satisfaction during a time of assisted reproduction between lesbian and heterosexual couples.

Table 1 displays the demographic data. There were no differences in age between participants (lesbian vs. heterosexual treated women p = 0.622; lesbian partners vs. heterosexual men p = 0.193); the majority were older than 30 years. Of treated women, 60% of lesbian and 65% of heterosexual were more than 30 years old. Of lesbian partners, 65% were older than 30 years and the corresponding age for heterosexual men was 76%.

**Table 1:** **Demographic data for women and men participating in the study**

		**Hetero**		**Lesbian**		
		**n**	**%**	**n**	**%**	p-value*
**Mother**						
Age	≤30	22	34.9	22	39.3	0.622
	>30	41	65.1	34	60.7	
Education	Elementary	0	0.0	1	1.8	0.375
	High school	23	36.5	16	28.1	
	University	40	63.5	40	70.2	
Biological children	No	55	100.0	53	94.6	0.243
	Yes	0	0.0	3	5.4	
Adoptive children	No	63	100.0	55	96.5	0.224
	Yes	0	0.0	2	3.5	
Step children	No	62	98.4	55	96.5	0.622
	Yes	41	65.1	34	60.7	
Child after treatment	No	30	47.6	17	29.8	0.046
	Yes	33	52.4	40	70.2	
**Co-mother/father**						
Age	≤30	15	24.2	20	35.1	0.193
	>30	47	75.8	37	64.7	
Education	Elementary	4	6.6	1	1.8	0.227
	High school	29	47.5	22	39.3	
	University	28	45.9	33	58.9	
Biological children	No	57	100.0	52	92.9	0.057
	Yes	0	0.0	4	7.1	
Adoptive children	No	63	100.0	57	100.0	-
	Yes					
Step children	No	59	93.7	57	100.0	0.121
	Yes	4	6.3	0	0.0	
Child after treatment	No	30	47.6	17	29.8	0.046
	Yes	33	52.4	40	70.2	

*Pearson’s Chi-square test. If cell count is below 5 Fisher’s exact test is used.

There were no differences in educational level between the couples.

Amongst the lesbian couples, 3 treated women and 4 partners had previous biological children; in the heterosexual couples there were no previous biological children.

More lesbian (70.2%) than heterosexual (52.4%) couples had had a successful assisted reproductive treatment resulting in the birth of a child (p = 0.046), see Table 1.

Satisfaction with relationship was assessed with the ENRICH inventory (Evaluating and Nurturing Relationship Issues, Communication and Happiness). Partner’s perception of relationship satisfaction is assessed in 10 dimensions each containing 10 items.

The scales are briefly described as follows:

- Personal issues: Examines an individual’s satisfaction with his or her partner’s behaviours.
- Communication: Is concerned with an individual’s feelings and attitudes towards communication in the relationship. Items focus on the level of comfort felt by the respondent in sharing and receiving emotional and cognitive information from the partner.
- Conflict resolution: Assesses the partner’s perception of the existence and resolution of conflict in the relationship. Items focus on how openly issues are recognised and resolved, as well as the strategies used to end arguments.
- Financial management: Focuses on the attitudes and concerns about the way economic issues are managed within the marriage/relationship. Items assess spending patterns and the manner in which financial decisions are made.
- Leisure activities: Assesses preferences for spending free time. Items reflect social versus personal activities, shared versus individual preferences, and expectations about spending leisure time as a couple.
- Sexual relationship: Examines the partner’s feelings about the affection and sexual relationship. Items reflect attitudes about sexual issues, sexual behaviour, and sexual fidelity.
- Children and parenting: Assesses attitudes and feelings about having and raising children. Items focus on decisions regarding discipline, goals for the children, and the impact of children in the couple’s relationship.
- Family and friends: Assesses feelings and concerns about relationships with relatives, in-laws, and friends. Items reflect expectations for and comfort with spending time with family and friends.
- Egalitarian roles: Focuses on an individual’s feelings and attitudes about various marital and family roles. Items reflect occupational, household, sex, and parental roles. High scores indicate a preference for more egalitarian roles.
- Conception of life: Examines the meaning of values, religious beliefs and practice, and conception of life within the marriage/relationship.

The Swedish version of this scale, originally created by Olson and co-workers [28] has been evaluated [29] and shown to be reliable and valid. Scores vary between 1 and 5, with higher scores indicating a better relationship. The scale has been used in long-term follow-up of IVF-couples and has been found to be a valid and reliable instrument to measure relationship satisfaction in clinical samples of couples requesting assisted reproduction [21-23].

The ENRICH inventory also assesses Positive Couples Agreement (PCA). Positive Couple Agreement is a measure of the couple’s congruence on each of the 10 dimensions. The partners’ responses are combined and the items that they agree on (within 1 point on a 1–5 scale) are summed and converted to a percentage score, which could range from 0 to 100%.

In 1993, Olson and Fowers [30] conducted an empirical typology based on ENRICH. 6267 couples participated. The study resulted in the identification of five distinct types of married couples. Vitalised couples reported high relationship quality in all subareas. Harmonious couples had relatively high relationship quality. Traditional couples had scores that were slightly above average with markedly higher scores on parenting and religious scales. Conflicted couples were characterised by moderately low scores on all but the role scale. The devitalised couples had the lowest scores on every ENRICH dimensions. The study did not report any mean values or cut-of scores for the different types of couples, however, the range reported for vitalised couples were mean 64.8–82.3 and for harmonious couples mean 42.1–64.8.

Initial analysis of the data included Pearson’s chi-square to evaluate the bivariate relationships between group (heterosexual and lesbian) and socio-demographic factors. The ENRICH scores (i.e. the ten factors as well as the total scores on both occasions) for the study group were tested for normality by use of the Kolmogorov-Smirnov test. As the assumption of normality could not be met in all of the studied variables, we chose to primarily use a non-parametric approach when analyzing the data. The Mann–Whitney U test was used to examine differences in ENRICH scores between the two study groups, as well as subgroups, while paired samples t-test was used to examine scores over time.

### Details of ethical approval

The study was designed according to the Helsinki declaration. On the 23 of February 2005, the Regional Ethical Review board in Linköping, Sweden approved the study, Dnr: M 29–05.

## Results

Tables 2 and 3 display results from the ENRICH assessment at commencement of treatment (T1) and at follow-up (T3). At T1, in general, lesbian couples reported better relationship satisfaction than heterosexual couples. The overall satisfaction with relationship quality decreased in both lesbian and heterosexual couples between T1 and T3. For treated lesbian women the results displayed a decrease in relationship satisfaction in all the ENRICH subareas except for Sexual Relationship and Family and Friends. The lesbian partners also displayed an overall decrease with exception for the subareas Sexual Relationship and Financial Management, Table 4.

**Table 2:** **The couples’ assessment of their relationship at acceptance for assisted reproduction**

	**Heterosexual**			**Lesbian**			Heterosexual vs.Lesbian*		
	**Mother**	**Father**	**All**	**Mother**	**Co-mother**	**All**	**Mother**	**Co-mother/father**	**all**
	**mean/SD**	**mean/SD**	**Mean/SD**	**mean/SD**	**mean/SD**	**Mean/SD**	**p-value**	**p-value**	**p-value**
Personality	43.8/4.5	41.9/4.8	42.9/4.7	44.7/4.4	45.1/3.7	44.9/4.0	0.169	<0.001	<0.001
Sexual	43.0/3.3	42.8/3.6	42.9/3.4	44.0/2.9	44.2/2.9	44.1/2.9	0.004	<0.001	<0.001
Children	43.6/3.7	43.9/3.4	43.8/3.6	44.7/2.6	44.5/2.7	44.6/2.6	0.029	0.004	<0.001
Family	44.1/4.1	43.0/5.0	43.5/4.5	45.1/3.9	45.6/3.8	45.4/3.9	0.158	0.121	0.038
Egalitarian	40.5/3.5	41.6/3.4	41.0/3.5	42.7/3.0	43.1/2.4	42.9/2.7	0.078	<0.001	<0.001
Conception	39.4/3.5	39.2/3.5	39.3/3.5	40.0/2.8	40.8/2.9	40.4/2.8	0.085	0.031	0.006
Communication	43.1/5.5	42.6/5.1	42.9/5.3	45.6/4.7	45.9/4.6	45.7/4.6	0.142	0.327	0.085
Conflict	40.5/5.7	39.3/5.9	39.9/5.8	42.7/5.3	42.4/5.01	42.5/5.2	0.155	0.001	0.001
Financial	43.0/4.1	42.7/4.2	42.8/4.12	43.9/4.4	43.7/4.7	43.8/4.6	<0.001	0.010	<0.001
Leisure	40.6/4.6	38.3/5.8	39.4/5.4	41.9/5.1	42.0/4.7	42.0/4.9	0.136	0.003	0.002
Total	421.6/30.4	415.3/32.0	418.5/31.2	435.3/28.3	437.3/25.8	436.3/27.0	0.009	<0.001	<0.001

*Mann–Whitney U-test.

**Table 3:** **The couples’ assessment of their relationship about three years after termination of assisted reproduction**

	**Heterosexual**				**Lesbian**			Heterosexual vs. Lesbian*	
	**Mother**	**Father**	**All**	**Mother**	**Co-mother**	**All**	**Mother**	**Co-mother/father**	**all**
	**mean/SD**	**mean/SD**	**Mean/SD**	**mean/SD**	**mean/SD**	**Mean/SD**	**p-value**	**p-value**	**p-value**
Personality	42.1/5.8	42.0/8.5	42.1/7.2	42.6/5.8	43.6/4.2	43.1/5.1	0.593	0.011	0.035
Sexual	41.6/9.3	41.8/9.9	41.7/9.6	42.1/5.8	43.2/5.4	42.6/5.6	0.412	0.026	0.034
Children	38.4/6.3	37.4/6.4	37.9/6.4	39.1/6.4	40.1/5.3	39.6/5.9	0.557	0.014	0.029
Family	42.6/4.9	42.4/7.0	42.5/6.0	42.4/4.9	42.8/4.3	42.6/4.6	0.744	0.708	0.977
Egalitarian	38.7/5.7	36.6/6.8	37.7/6.3	37.6/6.0	38.7/6.0	38.2/6.0	0.374	0.121	0.576
Conception	42.1/5.8	41.6/6.2	41.9/6.0	42.9/5.2	43.6/5.4	43.2/5.3	0.635	0.072	0.084
Communication	40.6/5.3	40.4/5.3	40.5/5.3	41.5/4.4	41.7/4.7	41.6/4.5	0.478	0.223	0.150
Conflict	42.2/4.4	42.3/6.4	42.3/5.5	43.8/4.6	43.7/5.0	43.8/4.8	0.037	0.038	0.003
Financial	38.4/4.6	40.1/3.9	39.2/4.3	40.1/4.8	41.1/4.0	40.6/4.4	0.041	0.100	0.007
Leisure	38.1/4.3	37.6/4.5	37.8/4.4	38.5/4.3	39.4/4.8	39.0/4.5	0.421	0.017	0.022
Total	404.9/42.0	402.2/46.2	403.6/44.0	410.6/41.3	417.9/37.5	414.3/39.4	0.480	0.035	0.041

*Mann–Whitney U-test.

**Table 4:** Test for difference on the ENRICH scores for each subscale comparing measurements before treatment and about three years after treatment/childbirth*

	**Heterosexual**		**Lesbian**		**Heterosexual**	**Lesbian**
	**Mother**	**Father**	**Mother**	**Co-mother**	**All**	**All**
	**p-value**	**p-value**	**p-value**	**p-value**	**p-value**	**p-value**
Personality	0.001	0.912	<0.001	0.007	0.150	<0.001
Sexual	0.145	0.124	0.117	0.348	0.034	0.072
Children	<0.001	<0.001	<0.001	<0.001	<0.001	<0.001
Family	0.002	0.389	0.054	0.002	0.009	<0.001
Egalitarian	<0.001	0.002	<0.001	<0.001	<0.001	<0.001
Conception	0.007	0.002	0.005	0.039	<0.001	0.001
Communication	0.127	0.467	<0.001	<0.001	0.123	<0.001
Conflict	0.001	<0.001	<0.001	<0.001	<0.001	<0.001
Financial	0.503	0.683	0.014	0.094	0.478	0.003
Leisure	0.002	0.066	<0.001	<0.001	0.001	<0.001
Total	<0.001	0.004	<0.001	<0.001	<0.001	<0.001

*Paired sample t-test.

In the heterosexual couples, women displayed a decrease in relationship satisfaction in the subareas Personality, Children, Family and Friends, Egalitarian Roles, Conception of Life, Conflict Resolution, Leisure Activities and Total ENRICH. Heterosexual men experienced a decrease in subareas Children, Egalitarian Roles, Conception of Life, Conflict resolution and Total ENRICH compared to T1, Table 4.

Table 5 displays results from the analysis of couple congruence.

**Table 5:** **The couples’ assessment of their relationship according to PCA (Positive Couple Agreement) at first treatment and about three years after termination**

	**Heterosexual**	**Lesbian**	Heterosexual vs. Lesbian*		**Heterosexual**	**Lesbian**	Hetero vs. Lesbian*
	**mean/SD**	**mean/SD**	**p-value**		**mean/SD**	**mean/SD**	**p-value**
Personality	66.3/20.5	80.9/17.0	<0.001	Personality	60.0/24.5	68.2/23.4	0.049
Sexual	84.4/16.2	88.4/15.4	0.057	Sexual	64.4/28.2	73.7/22.6	0.054
Children	74.9/17.1	79.6/11.0	0.137	Children	60.3/24.3	66.8/18.2	0.107
Family	74.6/19.1	81.9/16.7	0.009	Family	68.1/21.2	76.3/20.1	0.005
Egalitarian	64.3/16.5	75.1/12.3	<0.001	Egalitarian	56.3/21.3	64.6/20.1	0.014
Conception	34.3/15.1	70.7/8.4	0.004	Conception	57.3/19.0	63.5//18.0	0.008
Communication	71.9/22.3	85.3/17.4	<0.001	Communication	59.8/28.3	70.7/21.8	0.024
Conflict	57.5/22.8	68.1/21.2	0.003	Conflict	44.1/27.2	54.7/25.4	0.008
Financial	71.0/18.3	75.3/18.8	0.119	Financial	66.2/25.2	70.9/17.2	0.568
Leisure	57.9/21.5	68.6/24.3	0.001	Leisure	44.4/26.2	52.1/22.1	0.038
Total	687.1/143.9	773.9/118.1	<0.001	Total	581.1/198.5	661.6/165.5	0.011

*Mann–Whitney U-test.

**Figure 2:**
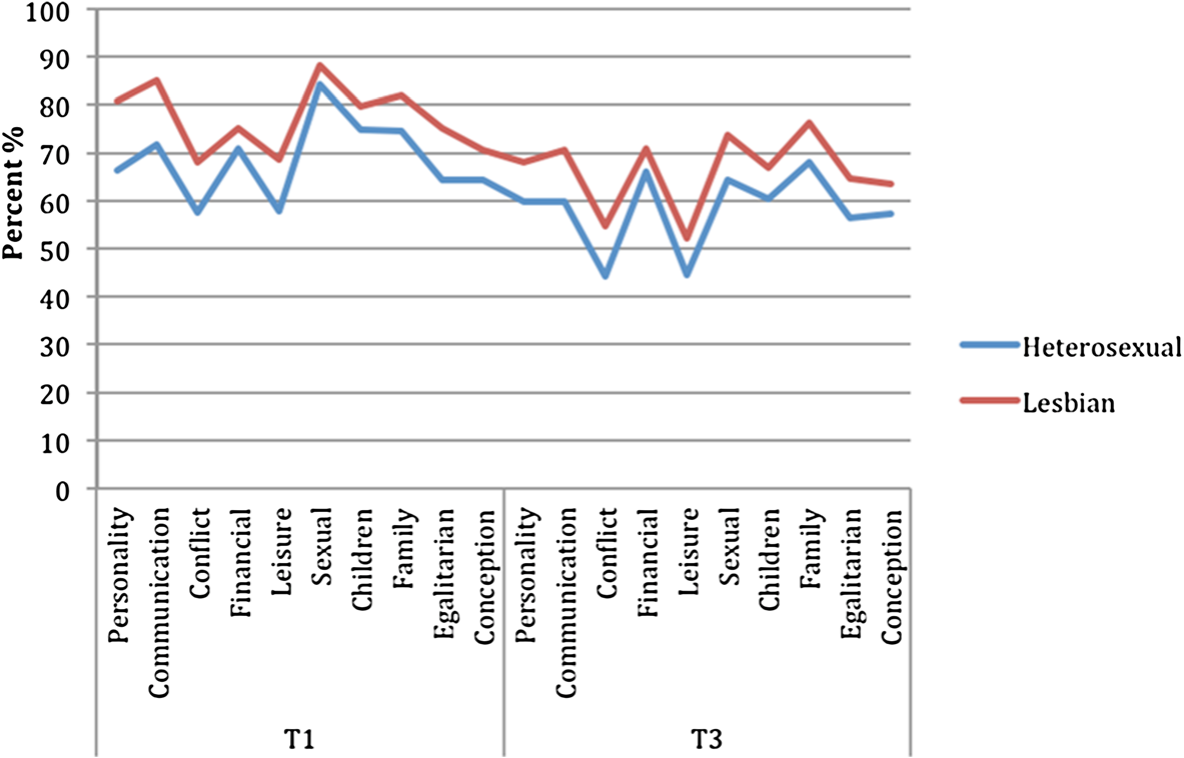
**Couples congruence on Enrich subscales at T1 and T3.**

At T1 as well as T3, the lesbian couples had higher congruence scores than the heterosexual couples on all subareas except for Children and Financial. Testing for differences on PCA-scores between measurements before treatment and at follow-up both lesbian and heterosexual couples reported a decrease in satisfaction in all relationship dimensions, Figure 2.

Difference on the ENRICH scores within the couples for each subarea comparing measurements before treatment and three years after treatment, reported by successful and unsuccessful treatment, was measured. The treatment outcome (T3) was related to lesbian couples’ relationship satisfaction only in the subarea Communication, treated lesbian women (p = 0.018) and lesbian partners (p = 0.001) with a successful treatment reported lower satisfaction than those whose treatment did not result in a child. In the heterosexual couples however, women reported lower satisfaction with relationship after a successful treatment on a number of subareas; Egalitarian Roles (p = 0.025), Conception of Life (p = 0.038), Communication (p = 0.001), Conflict Resolution (p = 0.046) and Financial Management (p = 0.045). Heterosexual men’s perceptions were not associated by the success of the treatment.

Also an analysis of differences on the ENRICH scores on each subarea, before treatment and about three years after treatment, reported by successful or unsuccessful treatment and couple type was performed. An unsuccessful treatment was associated with lower scores from heterosexual men compared to lesbian partners in the subarea Communication (p = 0.031). Comparing lesbian and heterosexual treated women with a successful treatment, scores from lesbian treated women were higher than from heterosexual women on Conflict Resolution (p = 0.029) and Financial Management (p = 0.023). Finally, comparing lesbian partners and heterosexual men with a successful treatment, lesbian partners reported more satisfaction with Leisure activities (p = 0.023).

Finally, we tested for differences on ENRICH and PCA scores reported by age and level of education, and differences on ENRICH scores were found amongst women with a high school degree. At T3, heterosexual women with a high school degree reported lower scores than treated lesbian women with a high school degree in the subareas Personality (p = 0.003), Communication 0.018), Sexual (p = 0.018), Egalitarian Roles (p = 0.018) and Total ENRICH (p = 0.024), but higher than treated lesbian women on subareas Communication (p = 0.018), Leisure (p = 0.032) and Children (p = 0.032).

The association of age on ENRICH scores were minor. For women, the differences that appeared were from the group aged > 30 year. At T1 treated lesbian women had higher scores on several of subareas as in Total ENRICH (430.7 vs. 420.4 p = 0.001). At T3, this association remained only in three subareas and Total ENRICH was (403.2 vs. 402.4 p = 0.036). Amongst the group of younger (≤ 30 years) partners, at T1 lesbian partners had higher scores on some of the subareas and Total ENRICH (439.6 vs 414.4 p = 0.019) than heterosexual men. This association did not remain at T3, Total ENRICH (p = 0.227).

## Discussion

### Main findings

The main finding in this study was that the lesbian couples reported higher satisfaction with their relationship during the trajectories of assisted reproduction. Although there were differences in many of the ENRICH assessments, the heterosexual couples did not report a low relationship satisfaction. Both the lesbian couples and the heterosexual couples reported scores within the two best-functioning categories according to Olson & Fowers (1993) i.e. Harmonious Couples and Vitalised Couples [30].

### Strength and limitations

To date no previous studies have compared relationship satisfaction in lesbian and heterosexual couples during the time of undergoing assisted reproduction in Sweden. The data from this study are unique and contribute important knowledge to the existing research on planned lesbian families. The strengths of the study design also include the prospective longitudinal method, which allows investigation in changes over time. The couples were recruited from the whole of Sweden, at all university clinics that perform sperm donation treatment and hence the study is comprised of a wide range of couples from both rural and urban areas. Furthermore the ENRICH inventory is a well-established instrument which is frequently used in studies of couples undergoing assisted reproduction [21-23]. The large sample of 120 couples responding to questionnaires at two time points provide further strengths to the study.

However, one must bear in mind that the couples in this study are a selected group of stable couples that went to a fertility clinic to conceive. Hence, the result from this study can only be generalised to couples that undergo assisted reproduction in a clinical setting. To lesbian couples for example, there are ways to conceive outside the clinical setting, such as private arrangements with a known or ‘stranger’ donor (a donor the couple found on the internet for example); an insecure and troublesome route to conception, far away from the stability and safe treatment fertility clinics offer [14,15].

Due to responses from only one partner in the couple, many of the couples that participated at T1 dropped-out at T3. This means that the sample of this study is comprised of couples that are still cohabiting or married approximately three years after the commencement of treatment.

### Interpretation

Both lesbian and heterosexual couples reported a decrease in relationship satisfaction compared to when they first commenced treatment. Similar to previous findings [21-23], the subarea sexual relationship was the only subarea that, jointly for the couples, did not decrease over time. Our findings suggest that, rather than being different, lesbian and heterosexual couples’ experiences of relationship satisfaction after assisted reproduction and childbirth are similar to each other. Previously, both Kurdek (2005) and Peplau and Fingerhut (2007) have reported more similarities than differences between same-sex and heterosexual couples with regards to aspects of relationship quality and wellbeing [1,27].

It has been reported that lesbian couples are more egalitarian in their roles and share household and childcare tasks differently than heterosexual couples [31]. In this study, we could not see any differences in satisfaction with egalitarian roles; all parties, lesbian treated women and partners, and heterosexual women and men experienced a decrease in egalitarian roles. Perhaps this mirrors the fact that this is a selected group of couples, highly motivated towards parenthood and with stable relationships.

Some interesting differences were found between the couples when the treatment was unsuccessful. Whilst the lesbian treated women and their partners only reported a decline in relationship satisfaction in the subarea communication, an unsuccessful treatment seemed to affect heterosexual treated women much more; several of the subareas were associated with lower scores. Maybe one explanation for this can be found in the fact that many lesbian couples when they build their family, take in turn to be the birthmother [16,32]. In this way the lesbian couples may perceive that they have another chance to have a child if the assisted reproduction treatment of one of the women in the couple is unsuccessful. For the heterosexual women the alternatives after unsuccessful IVF-treatment are limited to gamete donation, adoption or to live without children. Another suggestion to explain the influence an unsuccessful treatment had on heterosexual women’s perception of relationship satisfaction is that this might be an expression of pressure by social expectations to form a traditional nuclear family, to conceive and form a family with children.

Some minor differences emerged when the treatment was successful and resulted in the birth of a child. The subareas conflict and financial revealed a significant difference between the lesbian and heterosexual treated women, and the heterosexual women reported lower satisfaction on this matter. Heterosexual men reported lower satisfaction in personality and leisure compared to lesbian partners with a successful treatment. Perhaps this can be explained by gender differences and that lesbian couples might benefit from the presence of two women in the couple. Some authors suggest that lesbian couples may be able to operate more easily in terms of equality because partners in lesbian couples create their relationships without reference to traditional roles and come to their relationships with a history of being socialised into the same gender roles [33]. It has also been suggested that same-sex couples may be more effective than their heterosexual counterparts in their ability to navigate conflict [34] and to work harmoniously on joint tasks [35]. Some suggest further that women are better support providers than men, and that female partners providing better support can also explain the lower level of conflict in lesbian couple [36].

## Conclusion

At a three-year follow up after assisted reproduction with donated sperm, lesbian couples reported stable relationships and a high satisfaction with their relationship, also after an unsuccessful treatment. Compared to heterosexual IVF couples, lesbian couples reported higher satisfaction.
